# Supplementary material for: Content-rich biological network constructed by mining PubMed abstracts
Source: BMC Bioinformatics. 2004 Oct 8;5:147. doi: 10.1186/1471-2105-5-147 (PMC528731; doi:10.1186/1471-2105-5-147)
Supplement: Additional File 2 — The original results of the above study (non-essential files are deleted to keep the file size under the limit set by BMC bioinformatics). [file 1471-2105-5-147-S2.bz2 › chilibotAdditionalFile2/dip10/64ID7729427E223/html/TAF13_TBP.html]

 


 **TAF13** and **TBP** 
  
Found 6 abstracts in PubMed, retrieved 6.  
 

 What does Google say? 
 PDF only 
| .edu only 

---

**Interactive relationship** (e.g. stimulation, inhibition, etc)

**Neutral relationship**- Unexpectedly, these assembly experiments reveal that TAF11 and  **TAF13**  appear to provide the critical functional contacts with  **TBP**  during PIC assembly.  Ref: 12840001 EMBO J< MedlineTA>EMBO J,
- Additionally, since SPT3 has been shown to contact yeast  **TBP**  directly and is the likely homolog of human  **TBP**  associated factor TAFII18  [ **TAF13** ] , we propose that RSP5 hRPF1 and SPT3 establish a functional link between activated PR and GR and the general transcription apparatus.  Ref: 8649367 Mol Cell Biol, 1996

**Non-interactive relationship** (e.g. studied together, co-existance, homology, etc.)

- By in situ chromosomal hybridization, and by somatic cell and radiation hybrid analysis, we have determined the genomic position of the human genes encoding four TAFII subunits of TFIID  [ **TBP** ]  TAFII150, TAFII105, TAFII68, TAFII18  [ **TAF13** ] , the three subunits of TFIIA TFIIA35 and TFIIA19, both encoded by the same gene, and TFIIA12, CDK8, and SURB7.  Ref: 11441538 Somat Cell Mol Genet, 1999
- Despite the significant sequence similarity with TAFII18  [ **TAF13** ] , native hSPT3 is not a bona fide TAFII because it is not associated in vivo either with human  **TBP**  TFIID  [ **TBP** ]  or with a TFIID  [ **TBP** ]  related  **TBP**  free TAFII complex.  Ref: 9726987 J Biol Chem, 1998
